# A computationally optimized broadly reactive hemagglutinin vaccine elicits neutralizing antibodies against influenza B viruses from both lineages

**DOI:** 10.1038/s41598-023-43003-2

**Published:** 2023-09-23

**Authors:** Michael A. Carlock, Ted M. Ross

**Affiliations:** 1 Center for Vaccines and Immunology, University of Georgia, Athens, GA USA; 2 Department of Infectious Diseases, University of Georgia, Athens, GA USA; 3 Global Vaccine Development, Florida Research and Innovation Center, Cleveland Clinic, Port Saint Lucie, FL USA; 4 Department of Infection Biology, Lehner Research Institute, Cleveland Clinic, Cleveland, OH USA; 5 Present Address: Global Vaccine Development, Florida Research and Innovation Center, Cleveland Clinic, 9801 SW Discovery Way, Port Saint Lucie, FL 34987 USA

**Keywords:** Immunology, Vaccines

## Abstract

Influenza B viruses (IBV) can cause severe disease and death much like influenza A viruses (IAV), with a disproportionate number of infections in children. Despite moving to a quadrivalent vaccine to include strains from both the B/Victoria and B/Yamagata lineages, vaccine effectiveness rates continue to be variable and low in many past seasons. To develop more effective influenza B virus vaccines, three novel IBV hemagglutinin (HA) vaccines were designed using a computationally optimized broadly reactive antigen (COBRA) methodology. These IBV HA proteins were expressed on the surface of a virus-like particle (VLP) and used to vaccinate ferrets that were pre-immune to historical B/Victoria or B/Yamagata lineage viruses. Ferrets vaccinated with B-COBRA HA vaccines had neutralizing antibodies with high titer HAI titer against all influenza B viruses regardless of pre-immunization history. Conversely, VLPs expressing wild-type IBV HA antigens preferentially boosted titers against viruses from the same lineage and there was little-to-no seroprotective antibodies detected in ferrets with mismatched IBV pre-immune infections. Overall, a single IBV HA developed using the COBRA methodology elicited protective broadly-reactive antibodies against current and future drifted IBVs from both lineages.

## Introduction

Influenza viruses cause seasonal epidemics and occasional worldwide pandemics. In addition, severe infection with influenza viruses results in hundreds of thousands of deaths every year^1,2^. Prevention of influenza virus infection by vaccination is the best way to minimize severe cases. Two types of influenza, A and B, co-circulate in the human population. Influenza A viruses (IAV) are subdivided into subtypes based upon the surface hemagglutinin (HA or H) and neuraminidase (NA or N). Currently, H1N1 and H3N2 subtypes have co-circulated in people since 1968. Influenza B viruses (IBV) were first isolated in 1940 and, in some seasons, IBVs are the dominant type isolated from people during flu season, though this can vary regionally and throughout a season as IAV and IBV can peak at different times^1,2^. During the 1988–1989 influenza season, an IBV strain antigenically distinct to the vaccine strain, B/Victoria/2/1987 (B/VIC/87), was detected. And by the next season, half of the isolates matched this new strain, B/Yamagata/16/1988 (B/YAM/88)^3^. As a result, IBV strains were categorized based on whether they were Victoria-like (B/VIC) or Yamagata-like (B/YAM)^4–6^. Viruses from the two lineages co-circulated afterwards, but similar to H1N1 and H3N2 IAVs, one lineage would out compete the other each flu season. From 2001 to 2012, the lineage recommended as the vaccine strain component changed six times^7^ and the predominant influenza B lineage selected for the vaccine matched the dominant circulating IBV lineage ~ 50% of the time^8^. In 2012, representatives from both IBV lineages were recommended for a quadrivalent influenza vaccine that resulted in increased vaccine effectiveness against influenza B virus infections^9,10^. Despite this, vaccine effectiveness is variable from season to season ranging from 34 to 76%^9^. As a result, our group developed more broadly-reactive influenza B HA antigens using a layered consensus approach termed computationally optimized broadly reactive antigen (COBRA), as previously described for the generation of influenza A antigens^11–23^.

Others have demonstrated cross-lineage protection through stalk-directed antibodies. Chimeric HA (cHA) proteins, made with an IBV stalk and the head of exotic IAVs, were used in sequential vaccinations with different HA heads in order to induce stalk-reactive antibodies^24^. Mice were protected from lethal challenge against B/YAM and B/VIC viruses, but the mechanism of protection was through non-neutralizing antibodies that engage Fc-mediated effector functions. This method was expanded upon through a “mosaic” HA (mHA) approach in which instead of replacing the entire head domain of IBV with one from IAV, site-directed replacements with sequences from exotic HAs were introduced at the major antigenic sites in the head domain^25^. The B mHA vaccinations were able to prevent mortality in mice and reduce morbidity, but like the cHA vaccinations, the afforded protection was through non-neutralizing antibodies. While these platforms certainly have merit, the reliance on non-neutralizing antibodies that do not prevent infection can be a limitation for use in humans, especially in vulnerable populations where simply ameliorating disease may not be enough. And currently, a reliable correlate of protection for non-neutralizing antibodies in humans has yet to be established.

The COBRA IBV (B-COBRA) HA vaccines elicited protective antibodies against viruses from both lineages. Moreover, these B-COBRA HA vaccines were more effective than wild-type HA in a pre-immune animal model. Immunological imprinting can greatly dictate immune responses to future influenza A viruses^26^, but it is unclear how IBV imprinting affects future immune responses to IBV strains. In this report, we explored how the breath of pre-existing immune responses elicited by infection of influenza B viruses could shape subsequent vaccine-induced antibodies.

## Results

### Cross-reactive antibodies elicited by influenza B viruses

In an effort to better understand how anti-IBV derived antibodies reacted against other influenza B strains, a ferret antibody landscape experiment was conducted. 14 influenza B strains representing a time period from 1940 to 2017 were used to infect ferrets and, 30 days later, collected sera was tested for hemagglutination inhibition (HAI) activity against a panel of influenza B viruses (Table 1). Nine different B/YAM viruses that were used to infect ferrets elicited antibodies with high HAI activity against all B/YAM strains in the panel, but little-to-no HAI activity against B/VIC strains. Ferrets infected with B/VIC viruses had antibodies with high HAI activity against all the B/VIC strains in the panel. In addition, ferrets infected with the 3 older viruses (B/HK/01, B/MY/04, and B/BR/08) had seroprotective HAI titers (≥ 1:40) against B/YAM strains. These same 3 B/VIC viruses elicited HAI activity against B/Lee/40, as did the older strains of B/YAM (isolated from 1988 to 1999). Ferret sera collected from B/Lee/40 infected ferrets had a high HAI titer (1:5120) against the B/Lee/40 virus, but low HAI activity against the B/YAM viruses in the panel and little-to-no HAI activity against the B/VIC strains.

**Table 1:**
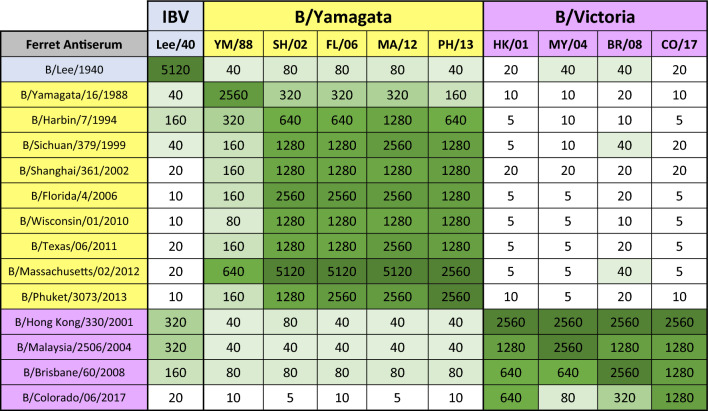
14 groups of ferrets (n = 2/group) were infected with different viruses spanning the IBV history in order to generate ferret reference sera.

The earliest IBV (B/Lee/1940) highlighted in blue, 9 viruses from the B/YAM lineage (1988–2013) highlighted in yellow, and 4 viruses from the B/VIC lineage (2001–2017) highlighted in purple. Serum was collected from each ferret, pooled per infection group, and then tested via HAI assay against 10 of the viruses (shown at the top).

### Characterization of IBV HA vaccines

**Figure 1:**
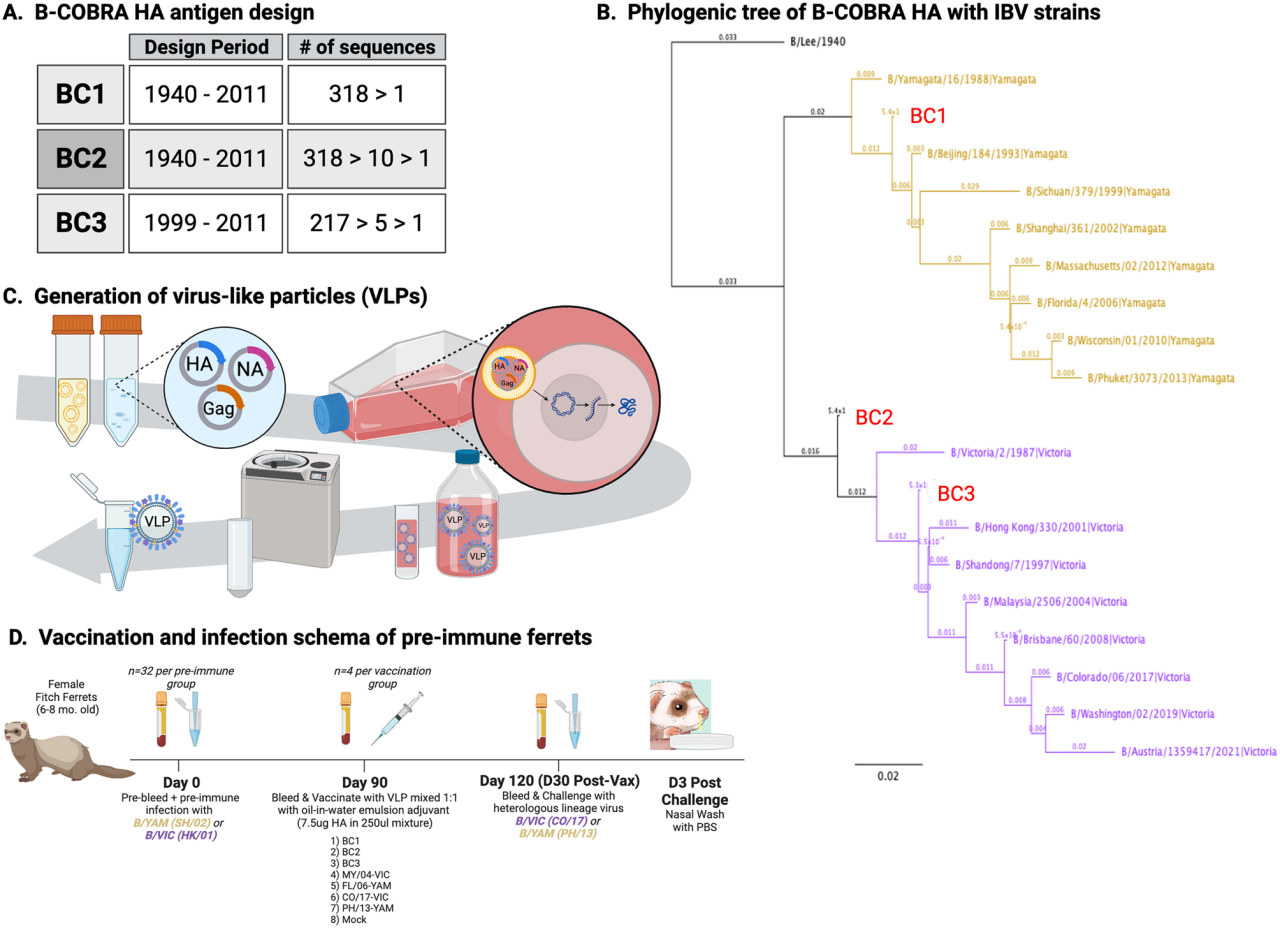
B-COBRA HA antigens for the vaccination and infection of pre-immune ferrets. (**A**) Publicly available HA sequences from 1940 to 2011 were downloaded and used to design three unique influenza B HA antigens: BC1, a single consensus sequence of 318 sequences during this period; BC2, the same 318 sequences antigenically layered into 10 secondary sequences and then a final consensus sequence; BC3, 217 sequences from 1999 to 2011 layered into 5 secondary sequences and then a final consensus sequence. (**B**) Phylogenetic analysis of B-COBRA candidates along with current and past IBV vaccine strains. HA1 regions were extracted and aligned using Muscle 3.8.425. The tree was rendered using FastTree 2.1.11. B/YAM lineage shown with yellow font, B/Victoria-lineage with violet font, and B-COBRA candidates in red font. (**C**) Three plasmids containing genes for HA, NA, or a Gag viral matrix protein were lipotransfected into mammalian 293T cells. After 3–5 days, supernatants were collected, centrifuged, filter-sterilized, and then ultracentrifuged with a 20% glycerol cushion. Pellets were resuspended in a small volume of PBS, and then HA activity and protein concentration were determined. (**D**) Immunologically naïve ferrets were infected intranasally (day 0) with a B/YAM (SH/02) or B/VIC (HK/01) virus and then vaccinated intramuscularly once (day 90) with one of three B-COBRA VLP vaccines (BC1, BC2, BC3), one of four VLP vaccines expressing wild-type HA proteins from the B/VIC lineage (MY/04 or CO/17) or B/YAM lineage (FL/06 or PH/13), or a mock vaccination containing PBS and adjuvant. All vaccines were adjuvanted 1:1 with a squalene-based emulsion adjuvant. Blood was collected from all animals at days 0, 90, and 120. All ferrets were challenged with an IBV heterologous to their pre-immune infection and nasal washes were collected 3 days post-infection. Created with BioRender.com.

Three initial hemagglutinin amino acid sequences were designed and characterized as vaccine candidates. HA sequences from 318 influenza B viruses isolated from 1940 to 2011 were aligned and the most common amino acid at each residue was determined resulting in an HA consensus sequence named BC1 (Fig. 1A). The same 318 IBV HA sequences were aligned using the multi-layered COBRA methodology and termed BC2. Another layered consensus alignment using 217 IBV HA amino acid sequences isolated from 1999 to 2011 was used to generate BC3. Phylogenetically, BC1 falls within the Yamagata-lineage, whereas BC2 and BC3 are aligned within or near the Victoria-lineage (Fig. 1B). For sequence alignments of these along with vaccine strains for IBV from 1940 to 2021, see Supplementary Fig. S1.

Immunological memory was established by infecting one set of ferrets with a B/YAM virus (B/SH/02) and a second set with a B/VIC virus (B/HK/01) prior to vaccination (Fig. 1D). These pre-immune ferrets were then vaccinated with virus-like particles (VLP) that were generated via lipotransfection into a mammalian cell line (Fig. 1C). The VLP vaccines consisted of one of the B-COBRA HA candidates or a wild-type HA from circulating influenza B viruses included in past commercial vaccines (Fig. 1D). Serum samples collected at day 90 (day of vaccination) following pre-immune infections, as well as sera collected at day 120 (~ 4 weeks post-vaccination), were tested for serological activity.

Influenza virus infection is initiated by viral HA binding to sialic acid receptors on the surface of host cells, and as such, anti-HA antibodies to block this initial binding is the best course of action in preventing viral infection^27,28^. The HAI assay mimicks this by measuring the highest dilution of anti-HA antibodies from serum that prevents hemagglutination from influenza virus binding to red blood cells^29^. In this study, we utilize the HAI assay to assess the functional activity of ferret antisera against a panel of 5 Yamagata-lineage viruses isolated from 1988 to 2013 and 5 Victoria-lineage viruses isolated from 1987 to 2019.

**Figure 2:**
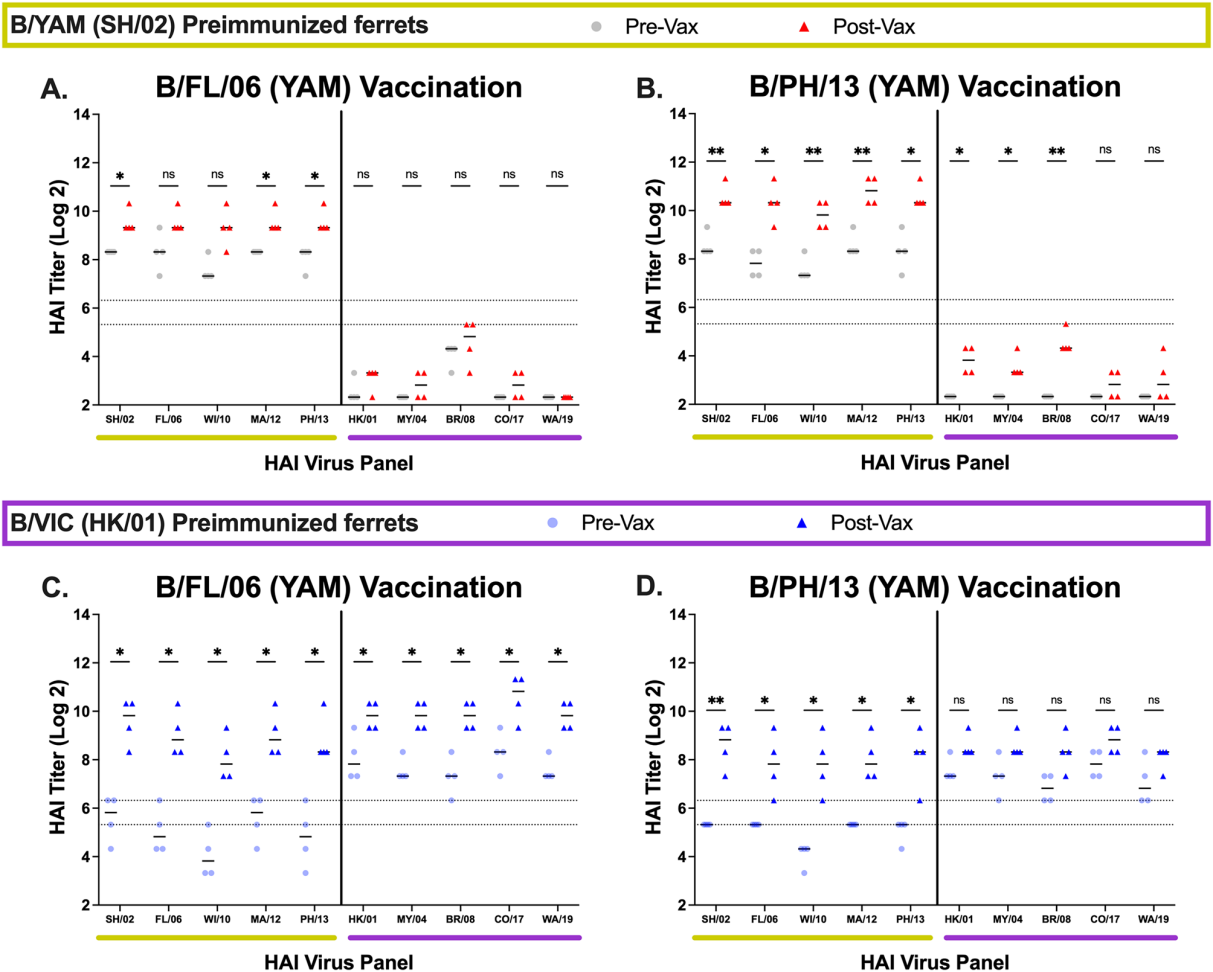
HAI serum antibody titers induced by wild-type B/YAM vaccinations. Immunologically naive ferrets were infected with a B/YAM virus, SH/02 (top), or with a B/VIC virus, HK/01 (bottom) and then vaccinated once 90 days later. Serum was collected at day 90 following preimmunization (Pre-Vax) and day 120 (Post-Vax). HAI titers were assessed against five B/YAM viruses (yellow) and five B/VIC viruses (purple). Values are the log2 HAI titers of each individual ferret (n = 4) from antisera collected at the two timepoints. Dotted lines indicate a 1:40 to 1:80 HAI titer range. Statistical analyses were performed using unpaired parametric t tests to determine significance of vaccine-induced antibodies: p > 0.05 = ns, p ≤ 0.05 = *, p ≤ 0.01 = **, p ≤ 0.001 = ***, p ≤ 0.0001 = ****.

Antisera from B/YAM (B/SH/02) infected ferrets that were vaccinated with VLP vaccines expressing wild-type B/YAM HA antigens derived from B/FL/06 or B/PH/13 (Fig. 2A,B) had > twofold increase in HAI activity on average against all 5 B/YAM strains following vaccination. These titers ranged between 1:80–1:320 prior to vaccination and 1:320–1:1280 post-vaccination. Little-to-no HAI activity against the B/VIC panel was observed. These same antigens used to vaccinate ferrets that were previously infected with B/VIC (B/HK/01) (Fig. 2C,D) elicited antibodies with high HAI titers against the entire panel. Sera with HAI activity against the B/YAM strains increased > eightfold from a titer of ~ 1:40 prior to vaccination. HAI titers to the B/VIC strains increased two- to fourfold from a pre-vaccination titer of ~ 1:160.

**Figure 3:**
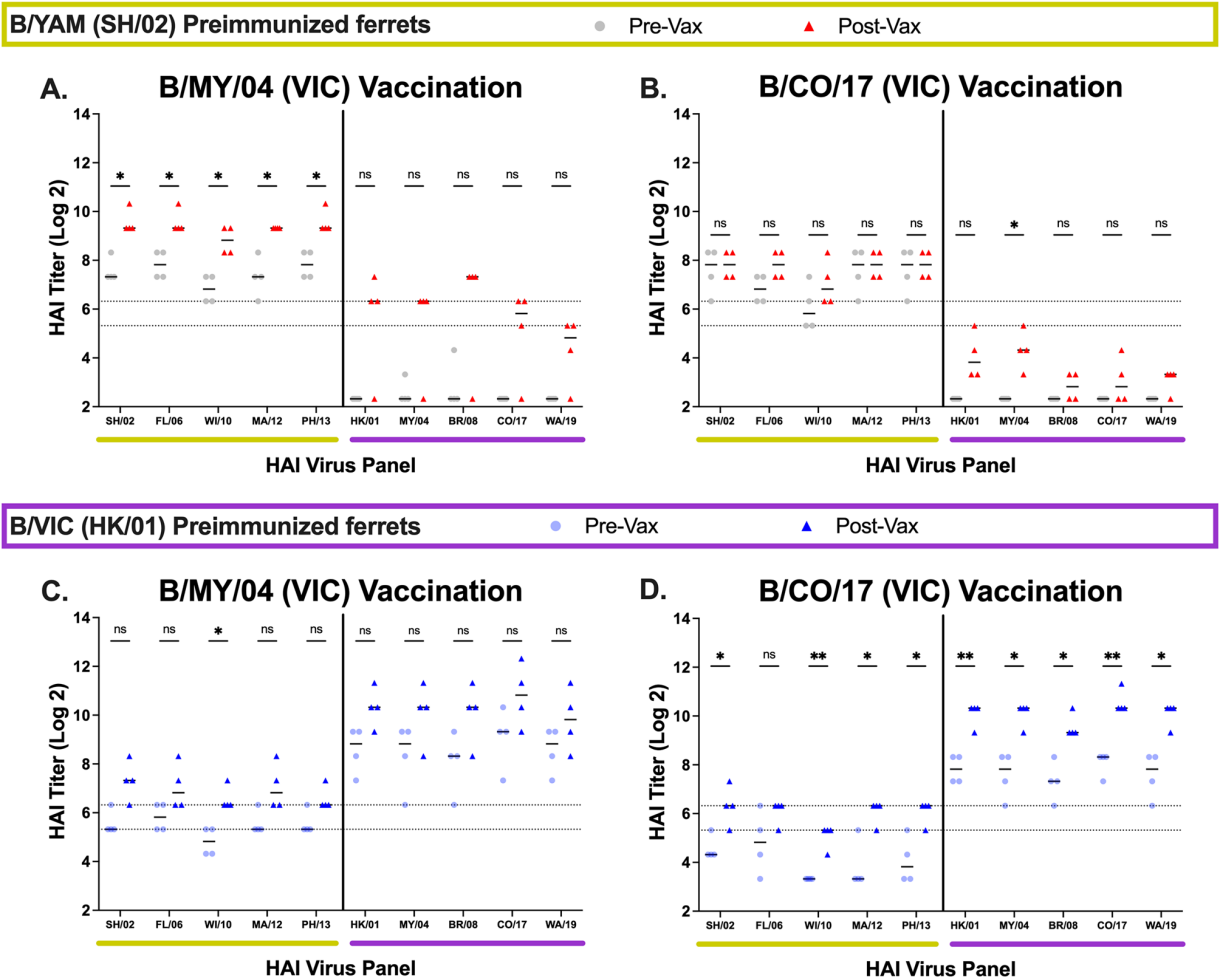
HAI serum antibody titers induced by wild-type B/VIC vaccinations. Immunologically naive ferrets were infected with a B/YAM virus, SH/02 (top), or with a B/VIC virus, HK/01 (bottom) and then vaccinated once 90 days later. Serum was collected at day 90 following preimmunization (Pre-Vax) and day 120 (Post-Vax). HAI titers were assessed against five B/YAM viruses (yellow) and five B/VIC viruses (purple). Values are the log2 HAI titers of each individual ferret (n = 4) from antisera collected at the two timepoints. Dotted lines indicate a 1:40 to 1:80 HAI titer range. Statistical analyses were performed using unpaired parametric t tests to determine significance of vaccine-induced antibodies: p > 0.05 = ns, p ≤ 0.05 = *, p ≤ 0.01 = **, p ≤ 0.001 = ***, p ≤ 0.0001 = ****.

Ferrets vaccinated with VLPs expressing the wild-type B/VIC HA from B/MY/04 or B/CO/17 (Fig. 3) had seroprotective HAI titers (≥ 1:40) with ~two- to fourfold increase against most of the viruses in the panel. These vaccinations in B/YAM pre-immune ferrets elicited lower HAI activity against B/VIC viruses (Fig. 3A,B). B/VIC pre-immune ferrets vaccinated with B/MY/04 had seroprotective antibody titers against all viruses in the panel, albeit they were not statistically significant (Fig. 3C). In contrast, B/VIC pre-immune ferrets vaccinated with B/CO/17 had higher HAI responses (~ fivefold increase) (Fig. 3D).

**Figure 4:**
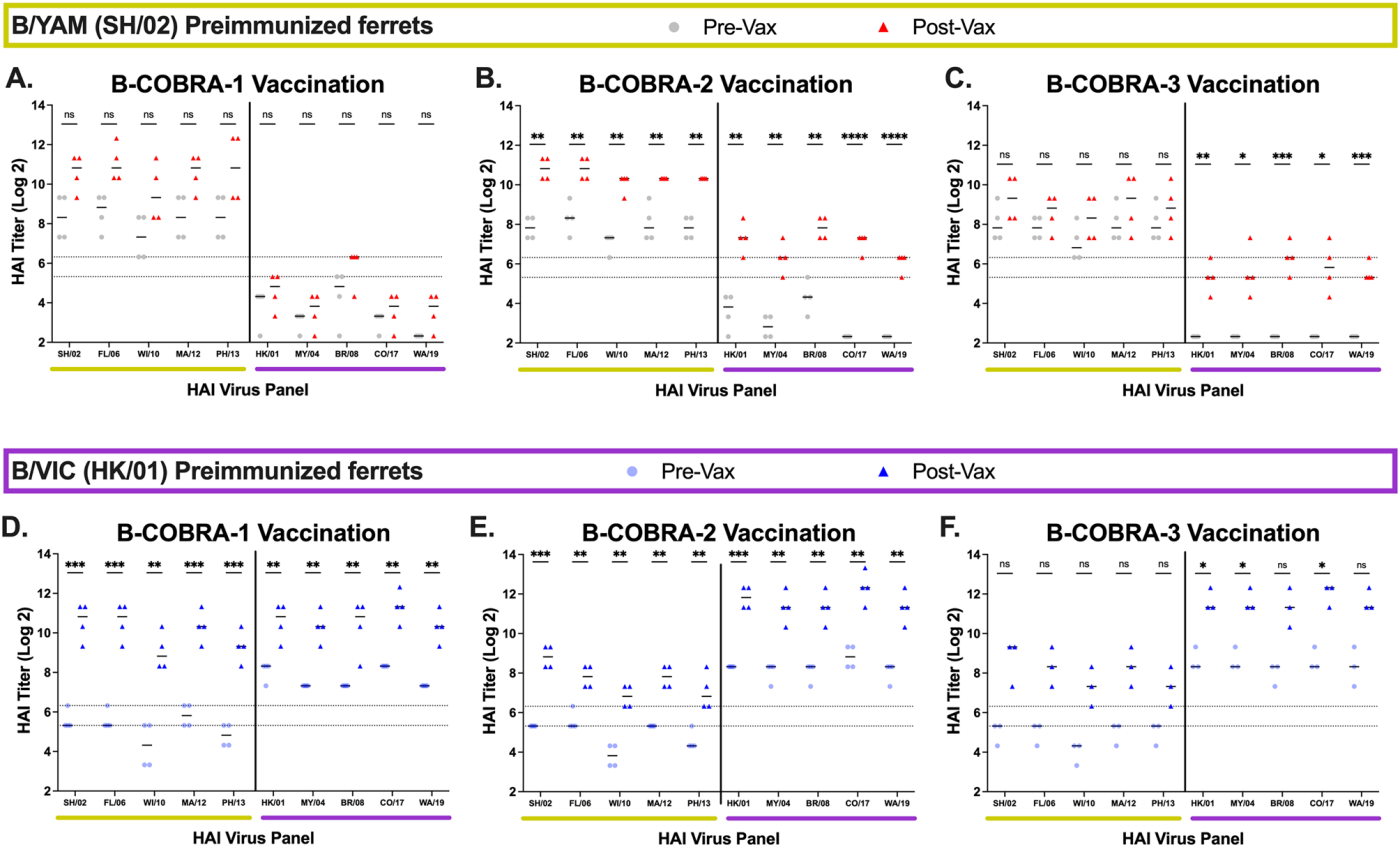
HAI serum antibody titers induced by B-COBRA HA vaccinations. Immunologically naive ferrets were infected with a B/YAM virus, SH/02 (top), or with a B/VIC virus, HK/01 (bottom) and then vaccinated once 90 days later. Serum was collected at day 90 following preimmunization (Pre-Vax) and day 120 (Post-Vax). HAI titers were assessed against five B/YAM viruses (yellow) and five B/VIC viruses (purple). Values are the log2 HAI titers of each individual ferret (n = 4^) from antisera collected at the two timepoints. Dotted lines indicate a 1:40 to 1:80 HAI titer range. Statistical analyses were performed using unpaired parametric t tests to determine significance of vaccine-induced antibodies: p > 0.05 = ns, p ≤ 0.05 = *, p ≤ 0.01 = **, p ≤ 0.001 = ***, p ≤ 0.0001 = **** [^one ferret lost from BC3 vaccinated group preimmunized with B/VIC].

Regardless of pre-immunization status, ferrets vaccinated with BC2 had a > sixfold increase in HAI activity against all the viruses in the panel. B/YAM pre-immune ferrets (Fig. 4B) had high HAI activity (~ 1:160) prior to vaccination against the B/YAM viruses and little-to-no HAI activity against the B/VIC viruses. Post-vaccination titers were boosted on average > sixfold (~ 1:1280) against the B/YAM viruses and 15-fold (~ 1:160) against the B/VIC viruses. B/VIC pre-immune ferrets (Fig. 4E) had HAI titers (~ 1:40) against the B/YAM viruses prior to vaccination and higher HAI activity (~ 1:320) against the B/VIC viruses. Post-vaccination titers for these ferrets were boosted sevenfold (~ 1:160) against the B/YAM viruses and tenfold (~ 1:2560) against the B/VIC panel of viruses. Similar HAI titers, albeit lower, were detected in BC3-vaccinated ferrets. No significant increases in HAI activity were observed against the B/YAM viruses in the B/YAM pre-immune ferrets, although all HAI titers exceeded seroprotective levels (Fig. 4C). The B/VIC pre-immune ferrets vaccinated with BC3 only had three ferrets in the group, thus reducing the significance scoring (a fourth point matching the others would have a p-value ≤ 0.01 against all viruses) (Fig. 4F).

Sera collected from BC1-vaccinated ferrets had similar, but generally higher HAI titers, compared to sera collected from ferrets vaccinated with wild-type B/YAM HA. B/YAM pre-immune ferrets vaccinated with VLPs expressing BC1 HA (Fig. 4A) had on average a > fivefold increase in HAI activity against the B/YAM strains following vaccination with HAI titers ranging ~ 1:160 prior to vaccination and 1:1280–1:2560 post-vaccination. Sera collected from these same ferrets had little-to-no HAI activity against the B/VIC viruses. The same antigens used in B/VIC pre-immune ferrets (Fig. 4D) elicited antibodies with high HAI titers against the entire panel of B/YAM strains with an increase of > 20-fold from a titer of ~ 1:40 prior to vaccination. These same ferrets had a five to eightfold increase in HAI titers against the B/VIC strains from a titer of ~ 1:160 prior to vaccination.

### Neutralization of influenza B viruses

**Figure 5:**
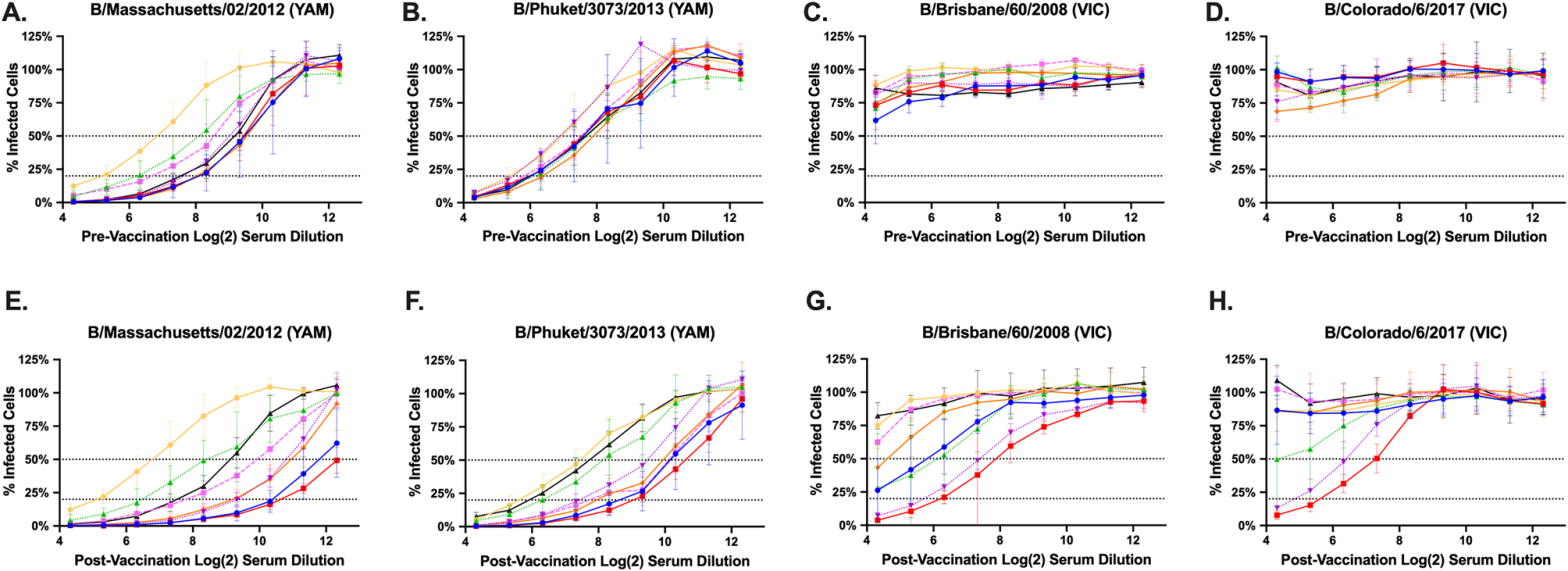
FRA neutralizing titers for Yamagata pre-immune ferrets vaccinated a single time with VLPs expressing different HA proteins: BC1, BC2, BC3, MY/04, FL/06, PH/13, CO/17, or Mock. Serum was collected at day 90 pre-vaccination (**A**–**D**) and day 120 post-vaccination (**E**–**H**) and tested against four IBVs: two from the Yamagata-lineage (**A**,**B**,**E**,**F**) and two from the Victoria-lineage (**C**,**D**,**G**,**H**). The dotted lines in the line graphs represent 50% inhibition and 80% inhibition of viral infection by antisera compared to virus-only control wells.

**Figure 6:**
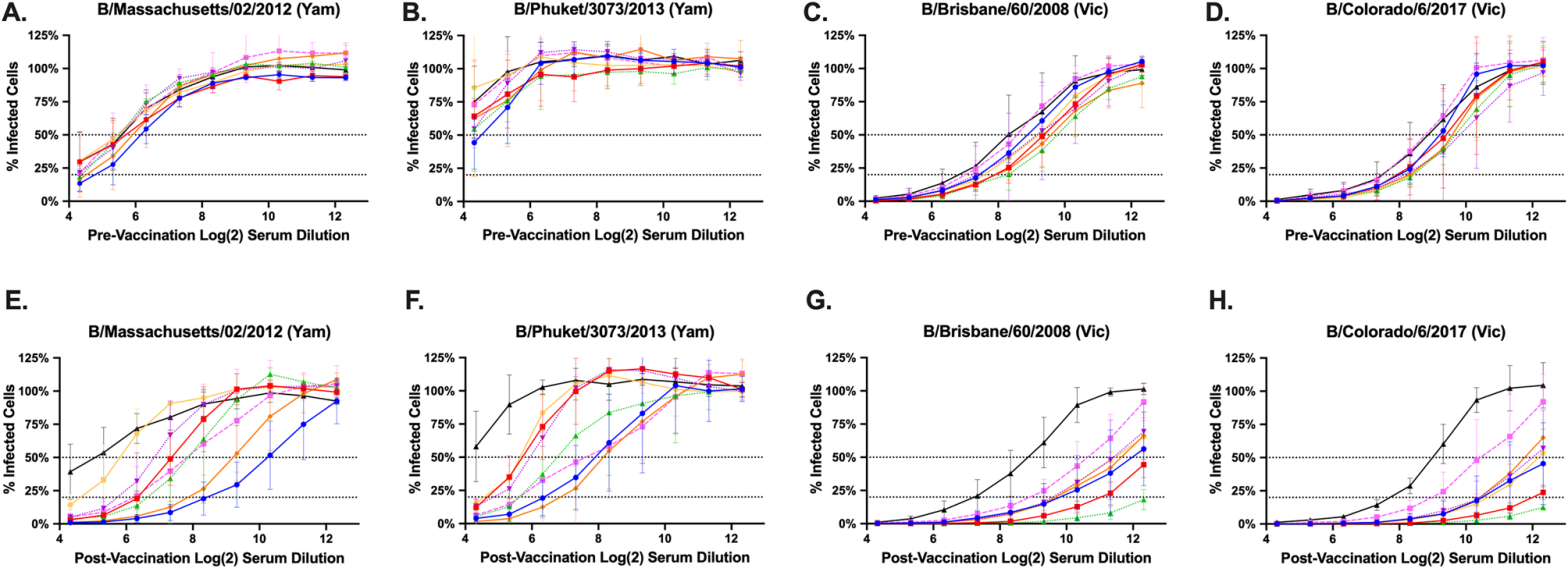
FRA neutralizing titers for Victoria pre-immune ferrets vaccinated a single time with VLPs expressing different HA proteins: BC1, BC2, BC3, MY/04, FL/06, PH/13, CO/17, or Mock. Serum was collected at day 90 pre-vaccination (**A**–**D**) and day 120 post-vaccination (**E**–**H**) and tested against four IBVs: two from the Yamagata-lineage (**A**,**B**,**E**,**F**) and two from the Victoria-lineage (**C**,**D**,**G**,**H**). The dotted lines in the line graphs represent 50% inhibition and 80% inhibition of viral infection by antisera compared to virus-only control wells.

While the HAI assay is still regarded as the “gold standard” for determining vaccine efficacy, microneutralization (MN) assays are often preferred amongst researchers since they are generally less subjective and can also capture other neutralizing antibodies that HAI does not, such as anti-stalk or NA-inhibiting antibodies^30^. Because the majority of neutralizing antibodies are anti-HA, however, MN and HAI generally correlate fairly well^31,32^, particularly when plaque reduction neutralization (PRNT) or focus reduction assay (FRA) are used which include ELISA as a readout^33,34^. In this study, we use FRA to evaluate the ability of serum antibodies to neutralize live virus infection for B/YAM (B/SH/02) pre-immunized ferrets (Fig. 5) and B/VIC (B/HK/01) pre-immunized ferrets (Fig. 6).

Ferrets infected with B/SH/02 virus had neutralizing antibodies against the two B/YAM viruses tested (Fig. 5A,B) with an FRA_50_ log_2_ titer > 6.8, but had no neutralizing antibodies against the two B/VIC viruses (Fig. 5C,D). VLP vaccinations boosted antibodies against the B/YAM viruses (Fig. 5E,F) to an FRA_50_ log_2_ titer ~ 10.0 for most vaccination groups. Ferrets vaccinated with either of the three B-COBRA HA candidates or the two older wild-type HA proteins (B/MY/04 and B/FL/06) had antisera that neutralized B/BR/08 infection (Fig. 5G). Only ferrets vaccinated with BC2, BC3, or B/MY/04 HA-expressing VLP vaccines were able to elicit neutralizing antibodies against B/CO/17 (Fig. 5H). For both B/VIC viruses, the highest vaccine-induced fold-change occurred in ferrets vaccinated with BC2 or B/MY/04 VLPs. For the B/YAM viruses, the highest vaccine-induced fold-change occurred in ferrets vaccinated with BC1 and BC2.

Ferrets pre-immunized with B/VIC (B/HK/01) had high neutralizing antibody titers against B/VIC viruses prior to vaccination (Fig. 6C,D) as well as cross-lineage neutralizing antibodies to B/MA/12 (Fig. 6A), but not to B/PH/13 (Fig. 6B). Ferrets vaccinated with BC1 HA VLPs or the B/YAM wild-type HA VLPs had the highest vaccine-induced increase in antibodies following the boost against the B/YAM viruses, with an FRA_50_ log_2_ titer of 7.83–10.26 to B/MA/12 (Fig. 6E) and 7.69–8.18 to B/PH/13 (Fig. 6F). Ferrets vaccinated with BC2 or BC3 HA VLPs had an FRA_50_ log_2_ titer of 7.39–7.86 against B/MA/12 and 5.74–6.77 against B/PH/13, whereas the B/VIC wild-type VLP vaccinated ferrets had titers of 5.82–6.85 against B/MA/12 and 5.66–5.97 against B/PH/13. All vaccinated ferrets had high post-vaccination titers (FRA_50_ log_2_ titer > 10) against the B/VIC viruses (Fig. 6G,H), with the highest titers recorded in ferrets vaccinated with BC2 or BC3 VLP vaccines.

### Heterologous challenge of pre-immune ferrets

**Figure 7:**
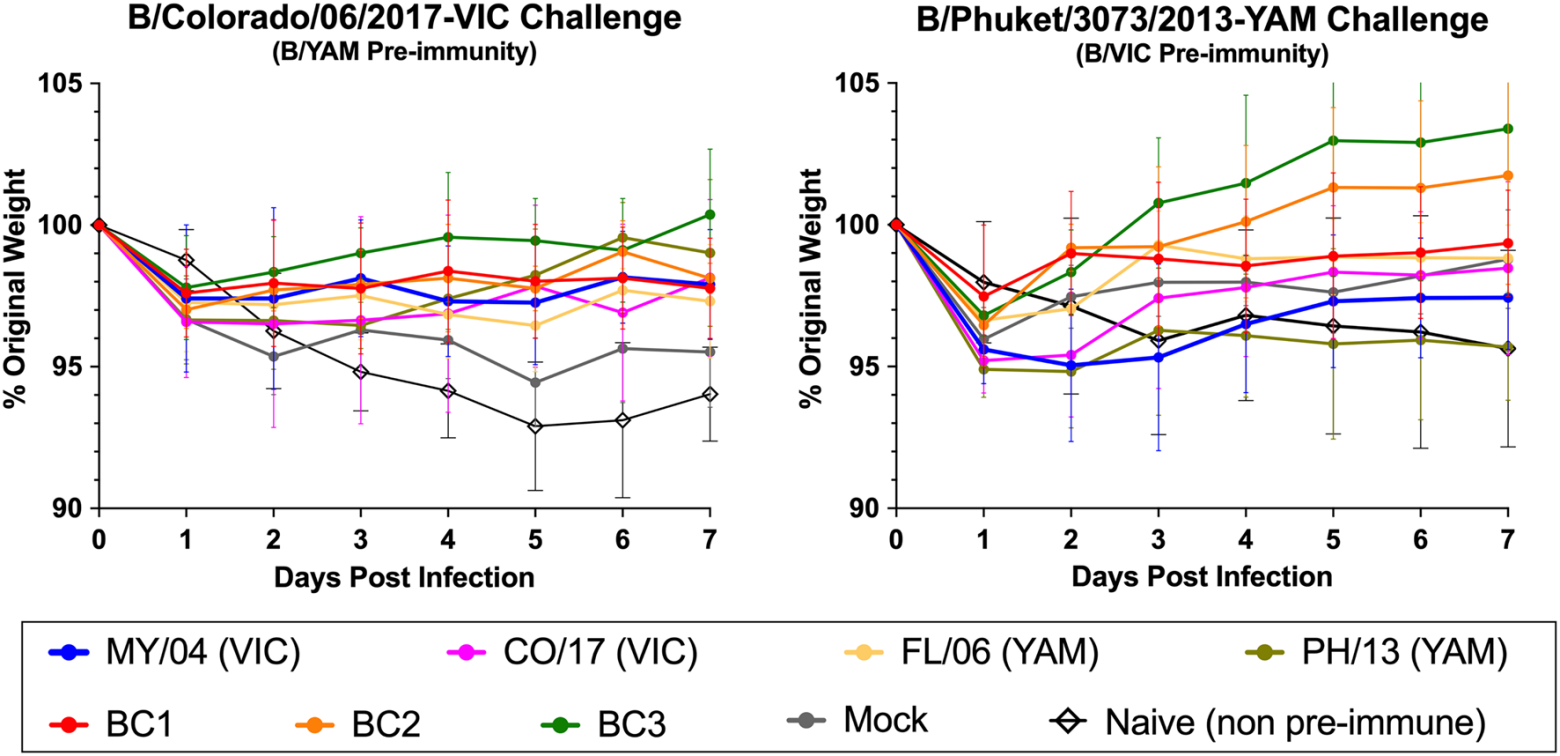
Average percent original weight of ferrets (n = 4 per group) following challenge with B/Colorado/06/2017-VIC (left) or B/Phuket/3073/2013-YAM (right). With exception to the naïve (non pre-immune and non-vaccinated) group of ferrets shown for comparison, all ferrets were previously infected with IBVs from lineages heterologous to the challenge strain (B/Shanghai/361/2002-YAM or B/Hong Kong/330/2001-VIC). Following these infections to establish pre-immunity, ferrets were allowed to sit for 90 days and then vaccinated a single time with VLPs expressing different HA proteins. Challenges were performed 30 days post-vaccination. Ferrets were monitored for weights and clinical symptoms daily, and nasal washes were collected 3 days post-infection.

All ferrets were challenged 30 days post-vaccination with a contemporary IBV from the B/VIC lineage (B/CO/17) or B/YAM lineage (B/PH/13) that were heterologous to the pre-immune infection virus (Fig. 7). Neither virus caused severe disease or death. The B/YAM pre-immune ferrets, as well as mock vaccinated ferrets, lost ~ 5% of their original body weight by day 5 following B/CO/17 challenge (Fig. 7A), which was slightly less than naïve, non-vaccinated ferrets (~ 7% average). All vaccinated ferrets had little weight loss and no signs of influenza virus infection over the time of observation. Comparable results were observed in B/VIC pre-immune ferrets that were challenged with B/PH/13 virus (Fig. 7B). Overall, all ferrets were protected against the influenza B virus challenge. Ferrets vaccinated with BC2 and BC3 HA antigens had less weight loss, on average, between the two different challenges and even gained weight over the period of observation (Fig. 7B). Little-to-no detectable viral titers were observed in nasal washes collected three days post-infection (data not shown).

## Discussion

Immunological imprinting or original antigenic sin following initial exposure to influenza has been well documented since it was first described in 1960^26^. This concept, however, has been less well described for influenza B virus infection, especially in regard to cross-lineage boosting or negative immunological interference. Using our pre-immune IBV ferret model, we further elucidate the impact of IBV induced pre-immunity on vaccine-induced antibody responses. Pre-immunization with a B/VIC virus led to an induction of low levels of cross-reactive antibodies and enabled B/YAM HA vaccinated ferrets to generate seroprotective antisera against B/VIC viruses. The same cross-reactivity was less apparent for B/VIC HA vaccinated ferrets pre-immunized to B/YAM, but some ferrets still generated seroprotective antibody levels. In homologously matched pre-immunization and vaccination regimens, ferrets receiving a B/YAM HA vaccination mounted a low response to B/VIC viruses. Ferrets vaccinated with the B/YAM-like BC1 HA vaccine elicited slightly higher titers than the wild-type B/YAM HA vaccinated ferrets, though the increases were not determined to be statistically significant. In contrast, almost all B/VIC pre-immune ferrets that were vaccinated with B/VIC HA had seroprotective titers to B/YAM viruses. B-COBRA HA vaccinations however elicited more broadly-reactive antibodies in these B/VIC pre-immunized ferrets. Following vaccination, all ferrets had seroprotective antibody titers against all IBVs in the panel and BC2 and BC3 vaccines efficiently elicited high titer antibodies in B/YAM pre-immune ferrets. All BC2 HA vaccinated ferrets had statistically significant increases in HAI titers against the full panel of IBVs and only one ferret in the BC3 group failed to mount a seroprotective HAI response to the B/VIC panel. Ferrets vaccinated with VLPs expressing wild-type vaccinations preferentially boosted antibody titers against lineage-matched viruses and generally did not have seroprotective titers, particularly for ferrets vaccinated with antigens heterologous to the pre-immunity. Both BC2 and BC3 HA vaccines elicited antibodies with broader HAI activity against a panel of IBVs than wild-type HA vaccine comparators.

Determining how initial exposure to one strain of influenza B virus impacts subsequent responses to future strains may be a key factor to consider when testing next-generation influenza vaccines, especially in children who may only have been exposed to an IBV strain from one lineage prior to vaccination. Other studies for example have shown that children vaccinated with a trivalent influenza vaccine (TIV) containing a B/YAM antigen elicited cross-reactive antibody responses against B/VIC viruses if they were previously immunized to B/VIC, but not if they were unexposed^35^. Data on B/VIC vaccinations in children with no pre-existing immunity to IBV is scarce, however children vaccinated with B/VIC based vaccines were shown to have low immune responses to B/VIC antigens when these children were primed with B/YAM the previous season^36^. Children immunized with vaccines containing B/VIC the following year had high anti-HA antibody titers to B/VIC, as well as cross-lineage back-boosting to the B/YAM HA antigens^36^.

Learning from imprinting is important—especially in regard to creating vaccines capable of “learning” from imprinting. More research is currently underway to better understand cellular mechanisms following vaccinations with COBRA HA, but we hypothesize that the inclusion of broader shared epitopes in antigen design leads to the recall of a broader B cell repertoire and increased breath of antibody response. For now, we show that B-COBRA HA vaccines were able to elicit serum antibodies in ferrets that had broader HAI activity and higher levels of neutralization against a panel of IBVs than wild-type comparators. Moreover, these broadly reactive influenza B antigens have the potential to replace the need for including both lineages in seasonal influenza vaccines, as well as better protection against future cross-lineage reassortments. That may not seem as relevant following the emergence of COVID-19, since no viruses in the B/YAM lineage have been detected since 2020^37–39^. It is possible that the lineage has become extinct, but it is too early to say for certain, especially since lineage-specific sequencing of IBV is not widely performed and PCR resources have been prioritized for SARS-CoV-2. It is also possible that Yamagata-like viruses could reappear as H1N1 did in 1977^38^. This would not be surprising given the extensive inter-lineage reassortment between the B/VIC and B/YAM lineages, and the regional specific and contained nature of IBVs. So having a broadly reactive antigen capable of neutralizing both lineages would be beneficial in preventing future reintroduction. An increased focus towards B/VIC epitopes may be warranted, but this may naturally occur for future B-COBRA HAs given that the COBRA methodology allows the natural evolution of influenza viruses to dictate the antigen design process^20^. These B-COBRA HA antigens utilized strains prior to 2012, and yet they were able to produce seroprotective titers against the future strains, often to similar levels as the matched modern wild-type HAs, and to higher levels when averaging the results from both pre-immune backgrounds. Therefore, these B-COBRA antigens have enormous potential at protecting against future drifted strains and will only be improved as next-generation COBRA HA antigens are developed that include more modern sequences.

Additional studies are also being conducted looking at glycosylation differences. Currently, most seasonal influenza vaccines are derived from propagating viruses in embryonated eggs. This, however, can result in egg adaptations that can cause reduced vaccine effectiveness due to changes in receptor binding^40^. For IBV, there is a potential glycosylation site in HA1 positions 197–199, located in a critical receptor binding region, where egg adaptations occur that eliminate this potential glycosylation^41^. Infections in people with IBVs isolated with this glycan have increased morbidity compared to infections with egg-adapted viruses without this glycan at this site^41,42^. Cell-derived viruses are more similar to naturally occurring viruses that humans encounter and therefore cell-derived vaccine antigens may be more effective at eliciting antibodies that neutralize influenza virus infections^42–46^. All the B-COBRA HA proteins retain this glycosylation site, as well as the B/PH/13 and B/CO/17 HA VLP vaccines used in this study, but the B/MY/04 and B/FL/06 HA VLPs do not. Moreover, all the viruses used for infections and serological analysis in this study were propagated in eggs and lacked this potential glycosylation. Since B-COBRA HA antigens were designed utilizing sequences from both IBV lineages, their final sequences included potential N-glycosylations present in both B/YAM and B/VIC strains. Another potential N-glycosylation site occurs in the 160-loop (HA1 162–167) located in a region that protrudes outward at the top of the HA^47^. As a result of insertions, this site is shifted by two amino acids for B/YAM viruses isolated after 2002. It was retained in B/VIC viruses, but in 2017, a series of amino acid deletions accumulated just before in HA1 162–164^48^. This deletion event led to vaccine strain changes for the B/VIC component, moving to a B/Colorado/06/2017-like strain (2-aa deletion) and then to a B/Washington/02/2019-like strain (3-aa deletion)^48^. Since these B-COBRA HA antigens were designed with sequences spanning until 2011, these deletions were not captured. Additional studies could determine the contribution of HA sequences with these amino acid deletions incorporated into future B-COBRA HA antigens, but the expectation would be improved protection against B/VIC viruses with a potential reduction in coverage to B/YAM viruses.

The advantages of our layering approach utilized with the COBRA methodology can be observed by comparing the BC1 and BC2 vaccinations. Both sequences were created with the same 318 HA sequences from IBVs isolated from 1940 to 2011. However, BC2, a product derived from employing our true approach to developing computationally optimized broadly reactive antigens, demonstrated remarkably better results. More work to assess the potential of these B-COBRA antigens needs to be completed, and current work is underway to help understand the mechanism on a cellular level. Regardless, these B-COBRA HA antigens and the COBRA methodology are certainly promising for improving seasonal influenza vaccine efficacy.

## Materials and methods

### Ferrets

Female fitch ferrets (*Mustela putorius furo*, 6–12 months of age, spayed and de-scented), negative for antibodies to circulating influenza B viruses, were purchased from Triple F Farms (Sayre, PA). Ferrets were pair-housed in stainless steel cages containing Sani-Chips laboratory animal bedding (P. J. Murphy Forest Products, Montville, NJ) and provided with Teklad Global Ferret Diet (Harlan Teklad, Madison, WI) and freshwater ad libitum. The University of Georgia Institutional Animal Care and Use Committee (IACUC) approved all experiments (A2021 06-016-Y3-A8) which were carried out at University of Georgia Animal Resources facilities in compliance with the ARRIVE guidelines. All methods were carried out in accordance with all relevant guidelines and regulations, including the National Research Council’s *Guide for the Care and Use of Laboratory Animals*, The Animal Welfare Act, and the CDC/NIH’s *Biosafety in Microbiological and Biomedical Laboratories* guide.

### Generation of ferret reference serum for IBV landscape

Ferrets (n = 2/group) were anesthetized using 3–5% vaporized isoflurane (Piramal Critical Care, Inc., Bethlehem, PA) and intranasally infected (~ 500 μL/nostril) with one of 14 influenza B viruses (10^4^–10^7^ PFU/mL) spanning the history of influenza B from 1940 to 2017. Animals were monitored daily during the infection for adverse events, including weight loss, loss of activity, nasal discharge, sneezing, and diarrhea. Two weeks later, ferrets were immunized subcutaneously above the footpad with 250 μL of concentrated virus. And then after another two weeks, blood was collected in serum separator tubes (SSTs) and antiserum was separated out and aliquoted into new tubes for use as reference material.

### Vaccine preparation

Mammalian 293T cells were transfected as previously described with three plasmids each containing genes in a mammalian-optimized expression vector: (1) an HA gene encoding IBV wild-type or COBRA-derived sequences, (2) a mismatched NA gene (A/mallard/Alberta/24/2001 H7N3), and (3) an unrelated viral matrix protein, HIV-1 Gag p55. After 96 h of incubation at 37 °C with 5% CO_2_, supernatants from transiently transfected cells were collected, centrifuged to remove any cellular debris, and then passed through a 0.22-μm filter. VLPs were then ultracentrifuged with a 20% glycerol cushion at 135,000 x g for 4 h at 4 °C and then resuspended in phosphate-buffered saline (PBS). Hemagglutination activity was confirmed for each VLP by serially diluting each in a 96-well V-bottom plate and then incubating for 30 min at room temperature with an equal volume of 0.8% turkey red blood cells (tRBCs). Total protein concentration was assessed by a conventional bicinchoninic acid assay (BCA).

### Viral infection and VLP vaccination of ferrets

Ferrets (n = 4/group) were anesthetized using 3–5% vaporized isoflurane (Piramal Critical Care, Inc., Bethlehem, PA) and then intranasally infected (~ 10^6^ PFU/mL with ~ 500 μL/nostril) with either an influenza B virus representing the Victoria lineage, B/Hong Kong/330/2001 (B/HK/01), or an influenza B virus representing the Yamagata lineage, B/Shanghai/361/2002 (B/SH/02). Animals were monitored daily during the infection for adverse events, including weight loss, loss of activity, nasal discharge, sneezing, and diarrhea. Ferrets were allowed to recover for 90 days and then, while anesthetized, bled and vaccinated once (250 μL) intramuscularly with VLP (7.5 μg HA) formulated 1:1 with an emulsified squalene-in-water AF03 adjuvant (Sanofi Pasteur, Lyon, France). 30 days later, ferrets were again anesthetized and bled, but then subsequently challenged (~ 10^8^ PFU/mL with ~ 500 μL/nostril) with modern IBVs heterologous to their initial pre-immune infection, either the Yamagata-like B/Phuket/3073/2013 (B/PH/13) or the Victoria-like B/Colorado/06/2017 (B/CO/17). Nasal washes were collected 3 days post-infection. Blood was collected in serum separator tubes (SSTs) at days 0, 90 (pre-vaccination), and 120 (D30 post-vaccination). Serum was separated, transferred to new tubes, and stored at 4 °C for use right away or frozen at − 20 °C for long-term storage.

### Viruses and HA antigens

Influenza viruses were obtained through the Influenza Reagents Resource (IRR), BEI Resources, the Centers for Disease Control (CDC), or were provided by Sanofi Pasteur and Virapur, LLC (San Diego, CA, USA). Viruses were passaged once in 10-day-old embryonated, specific-pathogen-free (SPF) chicken eggs per the protocol provided by the World Health Organization^49^. IBVs of the Victoria-lineage included the following strains: B/Hong Kong/330/2001 (B/HK/01), B/Malaysia/2506/2004 (B/MY/04), B/Brisbane/60/2008 (B/BR/08), B/Colorado/06/2017 (B/CO/17), and B/Washington/2/2019 (B/WA/19). IBVs of the Yamagata-lineage included the following strains: B/Yamagata/16/1988 (B/YM/88), B/Harbin/7/1994 (B/Har/94), B/Sichuan/379/1999 (B/Sic/99), B/Shanghai/361/2002 (B/SH/02), B/Florida/4/2006 (B/FL/06), B/Wisconsin/01/2010 (B/WI/10), B/Texas/06/2011 (B/TX/11), B/Massachusetts/02/2012 (B/MA/12), and B/Phuket/3073/2013 (B/PH/13). The first isolated IBV, B/Lee/1940 (Lee/40), was also included. All viruses were sequenced, and the HA sequences can be found in Supplementary Fig. S1.

### Hemagglutination-inhibition (HAI) assay

The HAI assay was used to assess functional antibodies to the HA that can inhibit agglutination of turkey erythrocytes. The protocols were adapted from the WHO Laboratory Influenza Surveillance Manual^49^. To inactivate non-specific inhibitors, sera were treated with a receptor-destroying enzyme (RDE) (Denka Seiken, Co., Japan) prior to being tested. Briefly, three parts of RDE were added to one part of sera and incubated overnight at 37 °C. RDE was inactivated by incubation at 56 °C for 30–45 min and then cooled to room temperature before diluting with 0.01 M PBS (pH 7.2; Gibco) to a final sera concentration of 1:10. RDE-treated sera were serially diluted in PBS two-fold across 96-well V-bottom microtiter plates. An equal volume (25 μl) of ether-treated influenza B virus^50^, adjusted beforehand via hemagglutination (HA) assay to a concentration of 8 hemagglutination units (HAU)/50 μl, was added to each well. The plates were covered and incubated at room temperature for 20 min, and then 0.8% turkey erythrocytes (Lampire Biologicals, Pipersville, PA, USA) in PBS were added. Red blood cells (RBCs) were prepared fresh each week, stored at 4 °C, and used within 72 h of preparation. The plates were mixed by agitation and covered, and the RBCs settled for 30 min at room temperature. The HAI titer was determined by the reciprocal dilution of the last well that contained non-agglutinated RBCs. Positive and negative serum controls were included for each plate. Seroprotection was defined as an HAI titer ≥ 1:40, seronegative with a titer less than 1:40, and seroconversion as a fourfold increase in titer compared to baseline resulting in a titer of ≥ 1:40^51^.

### Focal-reduction assay (FRA)

The FRA was initially developed by the WHO Collaborating Centre in London, modified by the CDC (Thomas Rowe, unpublished data), and previously described^52^. Briefly, MDCK-SIAT1 cells (250–300,000 cells/mL at 95–100% confluency) were added to 96-well F-bottom plates (100 μL/well) and incubated overnight at 37 °C with 5% CO_2_. The following day, cell monolayers were rinsed with 1 × PBS, and then RDE-treated serum was added (50 μL/well). Serum was serially diluted two-fold in virus growth medium with 1 μg/mL TPCK-trypsin (VGM-T) from a 1:20 starting dilution. Wild-type, non-treated virus (previously titrated and standardized to 600 FFU/50 μL) was then added (50 μL/well). Column 11 without sera served as the virus control (VC) and column 12 without sera or virus served as the cell control (CC). Plates were incubated for 2 h at 37 °C with 5% CO_2_ and then an overlay (modified Eagle medium with 0.1% BSA, 0.6% Avicel, 1μg/mL TPCK-Trypsin, and antibiotics) was added (100 μL/well). Plates were incubated for 18–22 h at 37 °C with 5% CO_2_ and then the overlay-virus mixtures were removed from each well. The cell monolayers were washed once with PBS and then fixed with ice-cold 4% formalin in PBS (100 μL/well) for 30 min at 4 °C. Plates were then washed twice with PBS and permeabilized with 0.5% Triton X-100 in PBS/glycine (100 μL/well) at room temperature for 25 min. The plates were washed three times with wash buffer (PBS with 0.05% Tween 20 or PBST) and then incubated for 1 h at RT with a monoclonal antibody (50 μL/well) against influenza B nucleoprotein (IRR, FR-52) diluted 1:2000 in ELISA buffer (PBS with 10% horse serum and 0.1% Tween 80). The plates were washed three times with PBST and then incubated 1 h at RT with goat anti-mouse peroxidase-labeled IgG (KPL, 474–1802) diluted 1:2000 in ELISA buffer. The plates were washed three times with PBST and then a TrueBlue substrate (SeraCare) containing 0.03% H_2_O_2_ was added (50 μL/well) for 10 min at RT. The colorimetric reaction was stopped by washing the plates with distilled water. Plates were dried well, and then infectious foci (spots) were visualized and counted using a BioSpot analyzer with ImmunoCapture 6.4.87 software (CTL, Shaker Heights, OH). The FRA titer was reported as the reciprocal of the highest dilution of serum corresponding to 50% focus reduction compared to the VC minus the CC, so long as the VC is 200–1600 spots, and the CC is less than 21 spots.

### Statistical analysis

Statistical analyses were performed using GraphPad Prism software. For HAI, paired nonparametric t tests were performed to determine the significance of vaccine-induced antibodies between timepoints against each strain for each pre-immune background. Additionally, 2way ANOVA analyses were performed to determine the significance between each vaccination group (data not shown). P values of ≤ 0.05 were considered significant.

### Supplementary Information

Supplementary Legends.

Supplementary Figure 1.

Supplementary Information 3.

Supplementary Information 4.

Supplementary Information 5.

Supplementary Information 6.

Supplementary Information 7.

## Data Availability

The datasets generated and/or analyzed during the current study are available in the NIAID/NIH CIVICs C3 website repository for review and download: https://www.niaidcivics.org/. In addition, HAI raw data available in Supplementary File S1. Values transformed to Log(2) in Prism for Figs. 1, 2 and 3. FRA results showing the average number of foci per vaccination group at each dilution divided by the average number of foci for the virus control, used to make Figs. 5 and 6, available in Supplementary File S2. Supplementary File S3 shows the FRA titer at 50% and 80% inhibition, calculated by finding the titer dilution at the point where each curve passes, or the estimated point if dilutions are higher than 12.32. Raw data from imager exports for individual ferrets available upon request. Weights in grams and percent original weight for individual ferrets shown in Supplementary File S4. Vaccination group averages and standard deviation used for Fig. 7 also shown. HA sequences for VLPs and egg-grown viruses shown in Supplementary File S5.
