# Supplementary material for: A computationally optimized broadly reactive hemagglutinin vaccine elicits neutralizing antibodies against influenza B viruses from both lineages
Source: Sci Rep. 2023 Sep 23;13:15911. doi: 10.1038/s41598-023-43003-2 (PMC10517972; doi:10.1038/s41598-023-43003-2)
Supplement: Supplementary file 1 — Supplementary Legends. [file 41598_2023_43003_MOESM1_ESM.docx]

**SUP FIG 1:** Sequence alignment of all viruses and VLPs used in study. Geneious Prime (2020.1.2) alignment view of Geneious Tree Builder (Jukes-Cantor genetic distance model with neighbor-joining tree build and B/Lee/1940 as an outgroup) shown following Geneious Alignment (Global Alignment with free end gaps, Blosum62 Cost Matrix). Amino acids that differ from the consensus sequence are highlighted.
