# Supplementary figures and images for: A computationally optimized broadly reactive hemagglutinin vaccine elicits neutralizing antibodies against influenza B viruses from both lineages
Source: Sci Rep. 2023 Sep 23;13:15911. doi: 10.1038/s41598-023-43003-2 (PMC10517972; doi:10.1038/s41598-023-43003-2)

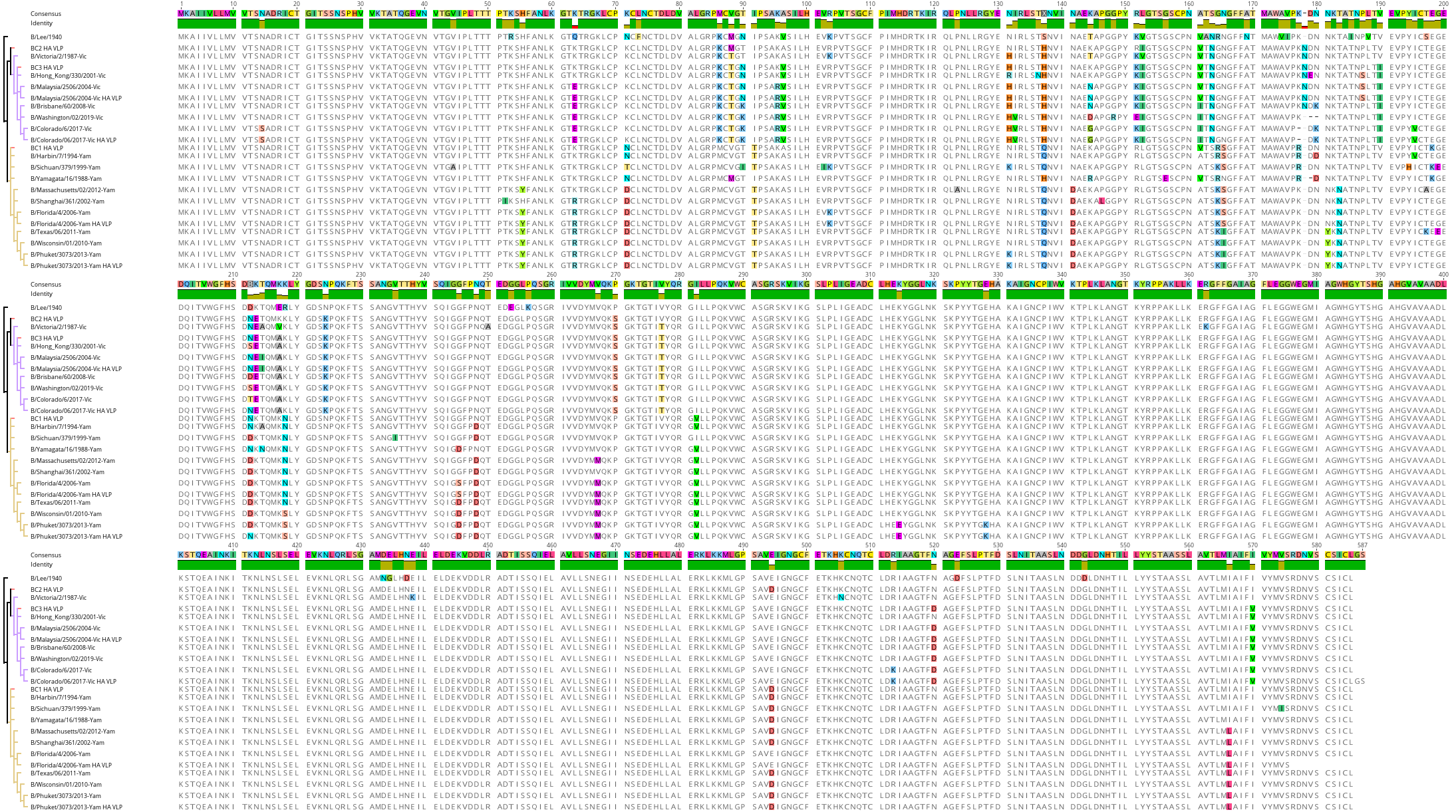

Supplement: Supplementary file 2 — Supplementary Figure 1. [file 41598_2023_43003_MOESM2_ESM.png]
