# Supplementary material for: A computationally optimized broadly reactive hemagglutinin vaccine elicits neutralizing antibodies against influenza B viruses from both lineages
Source: Sci Rep. 2023 Sep 23;13:15911. doi: 10.1038/s41598-023-43003-2 (PMC10517972; doi:10.1038/s41598-023-43003-2)
Supplement: Supplementary file 7 — Supplementary Information 7. [file 41598_2023_43003_MOESM7_ESM.docx]

**Supplementary File 5: IBV HA sequences for VLP vaccinations and live virus for serological assays**

**VLP SEQUENCES**

**B COBRA-1 HA VLP**

MKAIIVLLMVVTSNADRICTGITSSNSPHVVKTATQGEVNVTGVIPLTTTPTKSHFANLKGTKTRGKLCPNCLNCTDLDVALGRPMCVGTTPSAKASILHEVRPVTSGCFPIMHDRTKIRQLPNLLRGYENIRLSTQNVINAEKAPGGPYRLGTSGSCPNVTSRSGFFATMAWAVPRDNNKTATNPLTVEVPYICTKGEDQITVWGFHSDNKTQMKNLYGDSNPQKFTSSANGVTTHYVSQIGGFPNQTEDGGLPQSGRIVVDYMVQKPGKTGTIVYQRGVLLPQKVWCASGRSKVIKGSLPLIGEADCLHEKYGGLNKSKPYYTGEHAKAIGNCPIWVKTPLKLANGTKYRPPAKLLKERGFFGAIAGFLEGGWEGMIAGWHGYTSHGAHGVAVAADLKSTQEAINKITKNLNSLSELEVKNLQRLSGAMDELHNEILELDEKVDDLRADTISSQIELAVLLSNEGIINSEDEHLLALERKLKKMLGPSAVDIGNGCFETKHKCNQTCLDRIAAGTFNAGEFSLPTFDSLNITAASLNDDGLDNHTILLYYSTAASSLAVTLMIAIFIVYMVSRDNVSCSICL

**B COBRA-2 HA VLP**

MKAIIVLLMVVTSNADRICTGITSSNSPHVVKTATQGEVNVTGVIPLTTTPTKSHFANLKGTKTRGKLCPKCLNCTDLDVALGRPKCMGTIPSAKASILHEVRPVTSGCFPIMHDRTKIRQLPNLLRGYENIRLSTHNVINAEKAPGGPYRIGTSGSCPNVTNGNGFFATMAWAVPKNDNNKTATNPLTVEVPYICTEGEDQITVWGFHSDNETQMKKLYGDSKPQKFTSSANGVTTHYVSQIGGFPNQTEDGGLPQSGRIVVDYMVQKSGKTGTIVYQRGILLPQKVWCASGRSKVIKGSLPLIGEADCLHEKYGGLNKSKPYYTGEHAKAIGNCPIWVKTPLKLANGTKYRPPAKLLKERGFFGAIAGFLEGGWEGMIAGWHGYTSHGAHGVAVAADLKSTQEAINKITKNLNSLSELEVKNLQRLSGAMDELHNEILELDEKVDDLRADTISSQIELAVLLSNEGIINSEDEHLLALERKLKKMLGPSAVDIGNGCFETKHKCNQTCLDRIAAGTFNAGEFSLPTFDSLNITAASLNDDGLDNHTILLYYSTAASSLAVTLMIAIFIVYMVSRDNVSCSICL

**B COBRA-3 HA VLP**

MKAIIVLLMVVTSNADRICTGITSSNSPHVVKTATQGEVNVTGVIPLTTTPTKSHFANLKGTKTRGKLCPKCLNCTDLDVALGRPKCTGNIPSAKVSILHEVRPVTSGCFPIMHDRTKIRQLPNLLRGYEHIRLSTHNVINAEKAPGGPYKIGTSGSCPNVTNGNGFFATMAWAVPKNDNNKTATNPLTIEVPYICTEGEDQITVWGFHSDNETQMAKLYGDSKPQKFTSSANGVTTHYVSQIGGFPNQTEDGGLPQSGRIVVDYMVQKSGKTGTITYQRGILLPQKVWCASGRSKVIKGSLPLIGEADCLHEKYGGLNKSKPYYTGEHAKAIGNCPIWVKTPLKLANGTKYRPPAKLLKERGFFGAIAGFLEGGWEGMIAGWHGYTSHGAHGVAVAADLKSTQEAINKITKNLNSLSELEVKNLQRLSGAMDELHNEILELDEKVDDLRADTISSQIELAVLLSNEGIINSEDEHLLALERKLKKMLGPSAVEIGNGCFETKHKCNQTCLDRIAAGTFDAGEFSLPTFDSLNITAASLNDDGLDNHTILLYYSTAASSLAVTLMIAIFVVYMVSRDNVSCSICL

**B/Malaysia/2506/2004-Vic HA VLP**

MKAIIVLLMVVTSNADRICTGITSSNSPHVVKTATQGEVNVTGVIPLTTTPTKSHFANLKGTETRGKLCPKCLNCTDLDVALGRPKCTGNIPSARVSILHEVRPVTSGCFPIMHDRTKIRQLPNLLRGYEHIRLSTHNVINAENAPGGPYKIGTSGSCPNVTNGNGFFATMAWAVPKNDNNKTATNSLTIEVPYICTEGEDQITVWGFHSDNEIQMAKLYGDSKPQKFTSSANGVTTHYVSQIGGFPNQTEDGGLPQSGRIVVDYMVQKSGKTGTITYQRGILLPQKVWCASGRSKVIKGSLPLIGEADCLHEKYGGLNKSKPYYTGEHAKAIGNCPIWVKTPLKLANGTKYRPPAKLLKERGFFGAIAGFLEGGWEGMIAGWHGYTSHGAHGVAVAADLKSTQEAINKITKNLNSLSELEVKNLQRLSGAMDELHNEILELDEKVDDLRADTISSQIELAVLLSNEGIINSEDEHLLALERKLKKMLGPSAVEIGNGCFETKHKCNQTCLDRIAAGTFDAGEFSLPTFDSLNITAASLNDDGLDNHTILLYYSTAASSLAVTLMIAIFVVYMVSRDNVSCSICL

**B/Florida/4/2006-Yam HA VLP**

MKAIIVLLMVVTSNADRICTGITSSNSPHVVKTATQGEVNVTGVIPLTTTPTKSYFANLKGTRTRGKLCPDCLNCTDLDVALGRPMCVGTTPSAKASILHEVKPVTSGCFPIMHDRTKIRQLPNLLRGYENIRLSTQNVIDAEKAPGGPYRLGTSGSCPNATSKSGFFATMAWAVPKDNNKNATNPLTVEVPYICTEGEDQITVWGFHSDDKTQMKNLYGDSNPQKFTSSANGVTTHYVSQIGSFPDQTEDGGLPQSGRIVVDYMMQKPGKTGTIVYQRGVLLPQKVWCASGRSKVIKGSLPLIGEADCLHEKYGGLNKSKPYYTGEHAKAIGNCPIWVKTPLKLANGTKYRPPAKLLKERGFFGAIAGFLEGGWEGMIAGWHGYTSHGAHGVAVAADLKSTQEAINKITKNLNSLSELEVKNLQRLSGAMDELHNEILELDEKVDDLRADTISSQIELAVLLSNEGIINSEDEHLLALERKLKKMLGPSAVEIGNGCFETKHKCNQTCLDRIAAGTFNAGEFSLPTFDSLNITAASLNDDGLDNHTILLYYSTAASSLAVTLMLAIFIVYMVS

**B/Phuket/3073/2013-Yam HA VLP**

MKAIIVLLMVVTSNADRICTGITSSNSPHVVKTATQGEVNVTGVIPLTTTPTKSYFANLKGTRTRGKLCPDCLNCTDLDVALGRPMCVGTTPSAKASILHEVRPVTSGCFPIMHDRTKIRQLPNLLRGYEKIRLSTQNVIDAEKAPGGPYRLGTSGSCPNATSKIGFFATMAWAVPKDNYKNATNPLTVEVPYICTEGEDQITVWGFHSDNKTQMKSLYGDSNPQKFTSSANGVTTHYVSQIGDFPDQTEDGGLPQSGRIVVDYMMQKPGKTGTIVYQRGVLLPQKVWCASGRSKVIKGSLPLIGEADCLHEEYGGLNKSKPYYTGKHAKAIGNCPIWVKTPLKLANGTKYRPPAKLLKERGFFGAIAGFLEGGWEGMIAGWHGYTSHGAHGVAVAADLKSTQEAINKITKNLNSLSELEVKNLQRLSGAMDELHNEILELDEKVDDLRADTISSQIELAVLLSNEGIINSEDEHLLALERKLKKMLGPSAVDIGNGCFETKHKCNQTCLDRIAAGTFNAGEFSLPTFDSLNITAASLNDDGLDNHTILLYYSTAASSLAVTLMLAIFIVYMVSRDNVSCSICL

**B/Colorado/06/2017-Vic HA VLP**

MKAIIVLLMVVTSSADRICTGITSSNSPHVVKTATQGEVNVTGVIPLTTTPTKSHFANLKGTETRGKLCPKCLNCTDLDVALGRPKCTGKIPSARVSILHEVRPVTSGCFPIMHDRTKIRQLPNLLRGYEHVRLSTHNVINAEGAPGGPYKIGTSGSCPNITNGNGFFATMAWAVPDKNKTATNPLTIEVPYVCTEGEDQITVWGFHSDNETQMAKLYGDSKPQKFTSSANGVTTHYVSQIGGFPNQTEDGGLPQSGRIVVDYMVQKSGKTGTITYQRGILLPQKVWCASGRSKVIKGSLPLIGEADCLHEKYGGLNKSKPYYTGEHAKAIGNCPIWVKTPLKLANGTKYRPPAKLLKERGFFGAIAGFLEGGWEGMIAGWHGYTSHGAHGVAVAADLKSTQEAINKITKNLNSLSELEVKNLQRLSGAMDELHNEILELDEKVDDLRADTISSQIELAVLLSNEGIINSEDEHLLALERKLKKMLGPSAVEIGNGCFETKHKCNQTCLDKIAAGTFDAGEFSLPTFDSLNITAASLNDDGLDNHTILLYYSTAASSLAVTLMIAIFVVYMVSRDNVSCSICLGS

**EGG-GROWN VIRUSES**

**B/Lee/1940 (413-Lee40_EFC-050818BLEE40EP2)**

MKAIIVLLMVVTSNADRICTGITSSNSPHVVKTATQGEVNVTGVIPLTTTPTRSHFANLKGTQTRGKLCPNCFNCTDLDVALGRPKCMGNIPSAKVSILHEVKPVTSGCFPIMHDRTKIRQLPNLLRGYENIRLSTSNVINAETAPGGPYKVGTSGSCPNVANRNGFFNTMAWVIPKDNNKTAINPVTVEVPYICSEGEDQITVWGFHSDDKTQMERLYGDSNPQKFTSSANGVTTHYVSQIGGFPNQTEDEGLKQSGRIVVDYMVQKPGKTGTIVYQRGILLPQKVWCASGRSKVIKGSLPLIGEADCLHEKYGGLNKSKPYYTGEHAKAIGNCPIWVKTPLKLANGTKYRPPAKLLKERGFFGAIAGFLEGGWEGMIAGWHGYTSHGAHGVAVAADLKSTQEAINKITKNLNSLSELEVKNLQRLSGAMNGLHDEILELDEKVDDLRADTISSQIELAVLLSNEGIINSEDEHLLALERKLKKMLGPSAVEIGNGCFETKHKCNQTCLDRIAAGTFNAGDFSLPTFDSLNITAASLNDDDLDNHTILLYYSTAASSLAVTLMIAIFIVYMVSRDNVSCSICL

**B/Victoria/2/1987-Vic (417-Vic87_MAC-05142019VIC87EP2)**

MKAIIVLLMVVTSNADRICTGITSSNSPHVVKTATQGEVNVTGVIPLTTTPTKSHFANLKGTKTRGKLCPKCLNCTDLDVALGRPKCTGTIPSAKASILHEVKPVTSGCFPIMHDRTKIRQLPNLLRGYEHIRLSTHNVINAETAPGGPYKVGTSGSCPNVTNGNGFFATMAWAVPKNDNNKTATNPLTVEVPYICTEGEDQITVWGFHSDNEAQMVKLYGDSKPQKFTSSANGVTTHYVSQIGGFPNQAEDGGLPQSGRIVVDYMVQKSGKTGTITYQRGILLPQKVWCASGRSKVIKGSLPLIGEADCLHEKYGGLNKSKPYYTGEHAKAIGNCPIWVKTPLKLANGTKYRPPAKLLKEKGFFGAIAGFLEGGWEGMIAGWHGYTSHGAHGVAVAADLKSTQEAINKITKNLNSLSELEVKNLQRLSGAMDELHNKILELDEKVDDLRADTISSQIELAVLLSNEGIINSEDEHLLALERKLKKMLGPSAVEIGNGCFETKHNCNQTCLDRIAAGTFNAGEFSLPTFDSLNITAASLNDDGLDNHTILLYYSTAASSLAVTLMIAIFIVYMVSRDNVSCSICL

**B/Yamagata/16/1988-Yam (425-Yam88_VP-I1627A)**

MKAIIVLLMVVTSNADRICTGITSSNSPHVVKTATQGEVNVTGVIPLTTTPTKSHFANLKGTKTRGKLCPNCLNCTDLDVALGRPMCMGTIPSAKASILHEVRPVTSGCFPIMHDRTKIRQLPNLLRGYENIRLSTHNVINAERAPGGPYRLGTSESCPNVTSRNGFFATMAWAVPRDNKTATNPLTVEVPYICTKGEDQITVWGFHSDNKNQMKNLYGDSNPQKFTSSANGVTTHYVSQIGDFPNQTEDGGLPQSGRIVVDYMVQKPGKTGTIVYQRGVLLPQKVWCASGRSKVIKGSLPLIGEADCLHEKYGGLNKSKPYYTGEHAKAIGNCPIWVKTPLKLANGTKYRPPAKLLKERGFFGAIAGFLEGGWEGMIAGWHGYTSHGAHGVAVAADLKSTQEAINKITKNLNSLSELEVKNLQRLSGAMDELHNEILELDEKVDDLRADTISSQIELAVLLSNEGIINSEDEHLLALERKLKKMLGPSAVDIGNGCFETKHKCNQTCLDRIAAGTFNAGEFSLPTFDSLNITAASLNDDGLDNHTILLYYSTAASSLAVTLMIAIFIVYMVSRDNVSCSICL

**B/Harbin/7/1994-Yam (426-Harb94_VP-J1626A)**

MKAIIVLLMVVTSNADRICTGITSSNSPHVVKTATQGEVNVTGVIPLTTTPTKSHFANLKGTKTRGKLCPNCLNCTDLDVALGRPMCVGTTPSAKASILHEVRPVTSGCFPIMHDRTKIRQLPNLLRGYENIRLSTQNVINAEKAPGGPYRLGTSGSCPNATSRSGFFATMAWAVPRDDNKTATNPLTVEVPYVCTEGEDQITVWGFHSDNKAQMKNLYGDSNPQKFTSSANGVTTHYVSQIGGFPDQTEDGGLPQSGRIVVDYMVQKPGKTGTIVYQRGVLLPQKVWCASGRSKVIKGSLPLIGEADCLHEKYGGLNKSKPYYTGEHAKAIGNCPIWVKTPLKLANGTKYRPPAKLLKERGFFGAIAGFLEGGWEGMIAGWHGYTSHGAHGVAVAADLKSTQEAINKITKNLNSLSELEVKNLQRLSGAMDELHNEILELDEKVDDLRADTISSQIELAVLLSNEGIINSEDEHLLALERKLKKMLGPSAVDIGNGCFETKHKCNQTCLDRIAAGTFNAGEFSLPTFDSLNITAASLNDDGLDNHTILLYYSTAASSLAVTLMIAIFIVYMVSRDNVSCSICL

**B/Sichuan/379/1999-Yam (432-Sic99_VP-I1627B)**

MKAIIVLLMVVTSNADRICTGITSSNSPHVVKTATQGEVNVTGAIPLTTTPTKSHFANLKGTKTRGKLCPTCLNCTDLDVALGRPMCVGITPSAKASILHEIKPVTSGCFPIMHDRTKIRQLPNLLRGYEKIRLSTQNVINAEKAPGGPYRLGTSGSCPNATSKSGFFATMAWAVPRDNNKTATNPLTVEVPHICTKEEDQITVWGFHSDDKTQMKNLYGDSNPQKFTSSANGITTHYVSQIGGFPDQTEDGGLPQSGRIVVDYMVQKPGKTGTIVYQRGILLPQKVWCASGRSKVIKGSLPLIGEADCLHEKYGGLNKSKPYYTGEHAKAIGNCPIWVKTPLKLANGTKYRPPAKLLKERGFFGAIAGFLEGGWEGMIAGWHGYTSHGAHGVAVAADLKSTQEAINKITKNLNSLSELEVKNLQRLSGAMDELHNEILELDEKVDDLRADTISSQIELAVLLSNEGIINSEDEHLLALERKLKKMLGPSAVDIGNGCFETKHKCNQTCLDRIAAGTFNAGEFSLPTFDSLNITAASLNDDGLDNHTILLYYSTAASSLAVTLMIAIFIVYMISRDNVSCSICL

**B/Hong_Kong/330/2001-Vic (418-HK01_VP-F1629A)**

MKAIIVLLMVVTSNADRICTGITSSNSPHVVKTATQGEVNVTGVIPLTTTPTKSHFANLKGTKTRGKLCPKCLNCTDLDVALGRPKCTGNIPSAKVSILHEVRPVTSGCFPIMHDRTKIRQLPNLLRGYERIRLSNHNVINAEKAPGGPYKIGTSGSCPNVTNGNGFFATMAWAVPKNENNKTATNSLTIEVPYICTEGEDQITVWGFHSDSETQMAKLYGDSKPQKFTSSANGVTTHYVSQIGGFPNQTEDGGLPQSGRIVVDYMVQKSGKTGTITYQRGILLPQKVWCASGRSKVIKGSLPLIGEADCLHEKYGGLNKSKPYYTGEHAKAIGNCPIWVKTPLKLANGTKYRPPAKLLKERGFFGAIAGFLEGGWEGMIAGWHGYTSHGAHGVAVAADLKSTQEAINKITKNLNSLSELEVKNLQRLSGAMDELHNEILELDEKVDDLRADTISSQIELAVLLSNEGIINSEDEHLLALERKLKKMLGPSAVEIGNGCFETKHKCNQTCLDRIAAGTFNAGEFSLPTFDSLNITAASLNDDGLDNHTILLYYSTAASSLAVTLMIAIFVVYMVSRDNVSCSICL

**B/Shanghai/361/2002-Yam (436-SH02_MAC-06212019SH02EP2)**

MKAIIVLLMVVTSNADRICTGITSSNSPHVVKTATQGEVNVTGVIPLTTTPIKSHFANLKGTRTRGKLCPDCLNCTDLDVALGRPMCVGTTPSAKASILHEVRPVTSGCFPIMHDRTKIRQLPNLLRGYENIRLSTQNVIDAEKALGGPYRLGTSGSCPNATSKSGFFATMAWAVPKDNNKNATNPLTVEVPYICTEGEDQITVWGFHSDDKTQMKNLYGDSNPQKFTSSANGVTTHYVSQIGGFPDQTEDGGLPQSGRIVVDYMVQKPGKTGTIVYQRGVLLPQKVWCASGRSKVIKGSLPLIGEADCLHEKYGGLNKSKPYYTGEHAKAIGNCPIWVKTPLKLANGTKYRPPAKLLKERGFFGAIAGFLEGGWEGMIAGWHGYTSHGAHGVAVAADLKSTQEAINKITKNLNSLSELEVKNLQRLSGAMDELHNEILELDEKVDDLRADTISSQIELAVLLSNEGIINSEDEHLLALERKLKKMLGPSAVDIGNGCFETKHKCNQTCLDRIAAGTFNAGEFSLPTFDSLNITAASLNDDGLDNHTILLYYSTAASSLAVTLMLAIFIVYMVSRDNVSCSICL

**B/Malaysia/2506/2004-Vic (419-MY04_VP-G1626A)**

MKAIIVLLMVVTSNADRICTGITSSNSPHVVKTATQGEVNVTGVIPLTTTPTKSHFANLKGTETRGKLCPKCLNCTDLDVALGRPKCTGNIPSARVSILHEVRPVTSGCFPIMHDRTKIRQLPNLLRGYEHIRLSTHNVINAENAPGGPYKIGTSGSCPNVTNGNGFFATMAWAVPKNDNNKTATNSLTIEVPYICTEGEDQITVWGFHSDNEIQMAKLYGDSKPQKFTSSANGVTTHYVSQIGGFPNQTEDGGLPQSGRIVVDYMVQKSGKTGTITYQRGILLPQKVWCASGRSKVIKGSLPLIGEADCLHEKYGGLNKSKPYYTGEHAKAIGNCPIWVKTPLKLANGTKYRPPAKLLKERGFFGAIAGFLEGGWEGMIAGWHGYTSHGAHGVAVAADLKSTQEAINKITKNLNSLSELEVKNLQRLSGAMDELHNEILELDEKVDDLRADTISSQIELAVLLSNEGIINSEDEHLLALERKLKKMLGPSAVEIGNGCFETKHKCNQTCLDRIAAGTFDAGEFSLPTFDSLNITAASLNDDGLDNHTILLYYSTAASSLAVTLMIAIFVVYMVSRDNVSCSICL

**B/Florida/4/2006-Yam (437-FL06_VP-F1627B)**

MKAIIVLLMVVTSNADRICTGITSSNSPHVVKTATQGEVNVTGVIPLTTTPTKSYFANLKGTRTRGKLCPDCLNCTDLDVALGRPMCVGTTPSAKASILHEVKPVTSGCFPIMHDRTKIRQLPNLLRGYENIRLSTQNVIDAEKAPGGPYRLGTSGSCPNATSKSGFFATMAWAVPKDNNKNATNPLTVEVPYICTEGEDQITVWGFHSDDKTQMKNLYGDSNPQKFTSSANGVTTHYVSQIGSFPDQTEDGGLPQSGRIVVDYMMQKPGKTGTIVYQRGVLLPQKVWCASGRSKVIKGSLPLIGEADCLHEKYGGLNKSKPYYTGEHAKAIGNCPIWVKTPLKLANGTKYRPPAKLLKERGFFGAIAGFLEGGWEGMIAGWHGYTSHGAHGVAVAADLKSTQEAINKITKNLNSLSELEVKNLQRLSGAMDELHNEILELDEKVDDLRADTISSQIELAVLLSNEGIINSEDEHLLALERKLKKMLGPSAVEIGNGCFETKHKCNQTCLDRIAAGTFNAGEFSLPTFDSLNITAASLNDDGLDNHTILLYYSTAASSLAVTLMLAIFIVYMVSRDNVSCSICL

**B/Brisbane/60/2008-Vic (422-BR08_VP-F1627A)**

MKAIIVLLMVVTSNADRICTGITSSNSPHVVKTATQGEVNVTGVIPLTTTPTKSHFANLKGTETRGKLCPKCLNCTDLDVALGRPKCTGKIPSARVSILHEVRPVTSGCFPIMHDRTKIRQLPNLLRGYEHIRLSTHNVINAENAPGGPYKIGTSGSCPNITNGNGFFATMAWAVPKNDKNKTATNPLTIEVPYICTEGEDQITVWGFHSDDETQMAKLYGDSKPQKFTSSANGVTTHYVSQIGGFPNQTEDGGLPQSGRIVVDYMVQKSGKTGTITYQRGILLPQKVWCASGRSKVIKGSLPLIGEADCLHEKYGGLNKSKPYYTGEHAKAIGNCPIWVKTPLKLANGTKYRPPAKLLKERGFFGAIAGFLEGGWEGMIAGWHGYTSHGAHGVAVAADLKSTQEAINKITKNLNSLSELEVKNLQRLSGAMDELHNEILELDEKVDDLRADTISSQIELAVLLSNEGIINSEDEHLLALERKLKKMLGPSAVEIGNGCFETKHKCNQTCLDRIAAGTFDAGEFSLPTFDSLNITAASLNDDGLDNHTILLYYSTAASSLAVTLMIAIFVVYMVSRDNVSCSICL

**B/Wisconsin/01/2010-Yam (439-WI10_EFC-042018BWIS10EP1)**

MKAIIVLLMVVTSNADRICTGITSSNSPHVVKTATQGEVNVTGVIPLTTTPTKSYFANLKGTRTRGKLCPDCLNCTDLDVALGRPMCVGTTPSAKASILHEVRPVTSGCFPIMHDRTKIRQLPNLLRGYENIRLSTQNVIDAEKAPGGPYRLGTSGSCPNATSKIGFFATMAWAVPKDNYKNATNPLTVEVPYICTEGEDQITVWGFHSDDKTQMKSLYGDSNPQKFTSSANGVTTHYVSQIGDFPDQTEDGGLPQSGRIVVDYMMQKPGKTGTIVYQRGVLLPQKVWCASGRSKVIKGSLPLIGEADCLHEKYGGLNKSKPYYTGEHAKAIGNCPIWVKTPLKLANGTKYRPPAKLLKERGFFGAIAGFLEGGWEGMIAGWHGYTSHGAHGVAVAADLKSTQEAINKITKNLNSLSELEVKNLQRLSGAMDELHNEILELDEKVDDLRADTISSQIELAVLLSNEGIINSEDEHLLALERKLKKMLGPSAVDIGNGCFETKHKCNQTCLDRIAAGTFNAGEFSLPTFDSLNITAASLNDDGLDNHTILLYYSTAASSLAVTLMLAIFIVYMVSRDNVSCSICL

**B/Texas/06/2011-Yam (440-TX11_VP-J1626B)**

MKAIIVLLMVVTSNADRICTGITSSNSPHVVKTATQGEVNVTGVIPLTTTPTKSYFANLKGTRTRGKLCPDCLNCTDLDVALGRPMCVGTTPSAKASILHEVRPVTSGCFPIMHDRTKIRQLPNLLRGYENIRLSTQNVIDAEKAPGGPYRLGTSGSCPNATSKIGFFATMAWAVPKDNYKNATNPLTVEVPYICKEEEDQITVWGFHSDDKTQMKNLYGDSNPQKFTSSANGVTTHYVSQIGDFPDQTEDGGLPQSGRIVVDYMMQKPGKTGTIVYQRGVLLPQKVWCASGRSKVIKGSLPLIGEADCLHEKYGGLNKSKPYYTGEHAKAIGNCPIWVKTPLKLANGTKYRPPAKLLKERGFFGAIAGFLEGGWEGMIAGWHGYTSHGAHGVAVAADLKSTQEAINKITKNLNSLSELEVKNLQRLSGAMDELHNEILELDEKVDDLRADTISSQIELAVLLSNEGIINSEDEHLLALERKLKKMLGPSAVDIGNGCFETKHKCNQTCLDRIAAGTFNAGEFSLPTFDSLNITAASLNDDGLDNHTILLYYSTAASSLAVTLMLAIFIVYMVSRDNVSCSICL

**B/Massachusetts/02/2012-Yam (441-MA12_VP-G1621B)**

MKAIIVLLMVVTSNADRICTGITSSNSPHVVKTATQGEVNVTGVIPLTTTPTKSYFANLKGTKTRGKLCPDCLNCTDLDVALGRPMCVGTTPSAKASILHEVRPVTSGCFPIMHDRTKIRQLANLLRGYENIRLSTQNVIDAEKAPGGPYRLGTSGSCPNATSKSGFFATMAWAVPKDNNKNATNPLTVEVPYICAEGEDQITVWGFHSDDKTQMKNLYGDSNPQKFTSSANGVTTHYVSQIGGFPDQTEDGGLPQSGRIVVDYMMQKPGKTGTIVYQRGVLLPQKVWCASGRSKVIKGSLPLIGEADCLHEKYGGLNKSKPYYTGEHAKAIGNCPIWVKTPLKLANGTKYRPPAKLLKERGFFGAIAGFLEGGWEGMIAGWHGYTSHGAHGVAVAADLKSTQEAINKITKNLNSLSELEVKNLQRLSGAMDELHNEILELDEKVDDLRADTISSQIELAVLLSNEGIINSEDEHLLALERKLKKMLGPSAVDIGNGCFETKHKCNQTCLDRIAAGTFNAGEFSLPTFDSLNITAASLNDDGLDNHTILLYYSTAASSLAVTLMLAIFIVYMVSRDNVSCSICL

**B/Phuket/3073/2013-Yam (442-PH13_VP-G1611B)**

MKAIIVLLMVVTSNADRICTGITSSNSPHVVKTATQGEVNVTGVIPLTTTPTKSYFANLKGTRTRGKLCPDCLNCTDLDVALGRPMCVGTTPSAKASILHEVRPVTSGCFPIMHDRTKIRQLPNLLRGYEKIRLSTQNVIDAEKAPGGPYRLGTSGSCPNATSKIGFFATMAWAVPKDNYKNATNPLTVEVPYICTEGEDQITVWGFHSDDKTQMKSLYGDSNPQKFTSSANGVTTHYVSQIGDFPDQTEDGGLPQSGRIVVDYMMQKPGKTGTIVYQRGVLLPQKVWCASGRSKVIKGSLPLIGEADCLHEEYGGLNKSKPYYTGKHAKAIGNCPIWVKTPLKLANGTKYRPPAKLLKERGFFGAIAGFLEGGWEGMIAGWHGYTSHGAHGVAVAADLKSTQEAINKITKNLNSLSELEVKNLQRLSGAMDELHNEILELDEKVDDLRADTISSQIELAVLLSNEGIINSEDEHLLALERKLKKMLGPSAVDIGNGCFETKHKCNQTCLDRIAAGTFNAGEFSLPTFDSLNITAASLNDDGLDNHTILLYYSTAASSLAVTLMLAIFIVYMVSRDNVSCSICL

**B/Colorado/06/2017-Vic (423-CO17_MAC-01292019CO17EP1)**

MKAIIVLLMVVTSSADRICTGITSSNSPHVVKTATQGEVNVTGVIPLTTTPTKSHFANLKGTETRGKLCPKCLNCTDLDVALGRPKCTGKIPSARVSILHEVRPVTSGCFPIMHDRTKIRQLPNLLRGYEHVRLSTHNVINAEGAPGGPYKIGTSGSCPNITNGNGFFATMAWAVPDKNKTATNPLTIEVPYVCTEGEDQITVWGFHSDTETQMAKLYGDSKPQKFTSSANGVTTHYVSQIGGFPNQTEDGGLPQSGRIVVDYMVQKSGKTGTITYQRGILLPQKVWCASGRSKVIKGSLPLIGEADCLHEKYGGLNKSKPYYTGEHAKAIGNCPIWVKTPLKLANGTKYRPPAKLLKERGFFGAIAGFLEGGWEGMIAGWHGYTSHGAHGVAVAADLKSTQEAINKITKNLNSLSELEVKNLQRLSGAMDELHNEILELDEKVDDLRADTISSQIELAVLLSNEGIINSEDEHLLALERKLKKMLGPSAVEIGNGCFETKHKCNQTCLDKIAAGTFDAGEFSLPTFDSLNITAASLNDDGLDNHTILLYYSTAASSLAVTLMIAIFVVYMVSRDNVSCSICL

**B/Washington/02/2019-Vic (424-WA19_UGA-BWA19-20200305)**

MKAIIVLLMVVTSNADRICTGITSSNSPHVVKTATQGEVNVTGVIPLTTTPTKSHFANLKGTETRGKLCPKCLNCTDLDVALGRPKCTGKIPSARVSILHEVRPVTSGCFPIMHDRTKIRQLPNLLRGYEHVRLSTHNVINAEDAPGRPYEIGTSGSCPNITNGNGFFATMAWAVPKNKTATNPLTIEVPYICTEGEDQITVWGFHSDSETQMAKLYGDSKPQKFTSSANGVTTHYVSQIGGFPNQTEDGGLPQSGRIVVDYMVQKSGKTGTITYQRGILLPQKVWCASGRSKVIKGSLPLIGEADCLHEKYGGLNKSKPYYTGEHAKAIGNCPIWVKTPLKLANGTKYRPPAKLLKERGFFGAIAGFLEGGWEGMIAGWHGYTSHGAHGVAVAADLKSTQEAINKITKNLNSLSELEVKNLQRLSGAMDELHNEILELDEKVDDLRADTISSQIELAVLLSNEGIINSEDEHLLALERKLKKMLGPSAVEIGNGCFETKHKCNQTCLDRIAAGTFDAGEFSLPTFDSLNITAASLNDDGLDNHTILLYYSTAASSLAVTLMIAIFVVYMVSRDNVSCSICL
